# Validity and reliability of innovative field measurements of tibial accelerations and spinal kinematics during cricket fast bowling

**DOI:** 10.1007/s11517-021-02381-3

**Published:** 2021-06-26

**Authors:** Billy Senington, Raymond Y. Lee, Jonathan Mark Williams

**Affiliations:** 1 School of Biosciences and Medicine, University of Surrey, Guildford, GU2 7WG UK; 2 Faculty of Technology, Portsmouth University, Portsmouth, UK; 3 Faculty of Health and Social Sciences, Bournemouth University, Bournemouth Gateway Building, St Paul’s Lane, Bournemouth, BH8 8GP Dorset UK

**Keywords:** Inertial sensors, Reliability, Accelerometer, Spine, Tibia

## Abstract

The use of inertial sensors in fast bowling analysis may offer a cheaper and portable alternative to current methodologies. However, no previous studies have assessed the validity and reliability of such methods. Therefore, this study aimed to assess the validity and reliability of collecting tibial accelerations and spinal kinematics using inertial sensors during in vivo fast bowling. Thirty-five elite male fast bowlers volunteered for this study. An accelerometer attached to the skin over the tibia was used to determine impacts and inertial sensors over the S1, L1 and T1 spinous processes used to derive the relative kinematics. These measurements were compared to optoelectronic and force plate data for validity analysis. Most acceleration and kinematics variables measured report significant correlations > 0.8 with the corresponding gold standard measurement, with intraclass correlation coefficients greater than 0.7. Low standard error of measurement and consequently small minimum detectable change (MDC) values were also observed. This study demonstrates that inertial sensors are as valid and reliable as current methods of fast bowling analysis and may provide some advantages over traditional methods. The novel metrics and methods described in this study may aid coaches and practitioners in the design and monitoring of fast bowling technique.

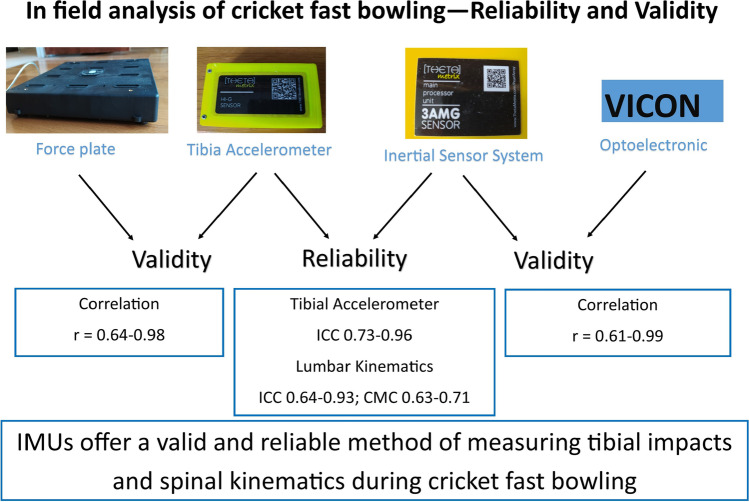

Graphical abstract illustrating the synopsis of the findings from this paper.

## Introduction

As a sport, cricket is not without its injuries, with 44% of injuries attributable to the fast bowlers [42]. The impact of these injuries is often long-lasting where they account for at least double the amount of cricket missed compared to any other injury [32]. Prevalence estimates suggest that low back injuries may be as high as 67%, with the fast bowling technique believed to offer some explanation for the high injury risk [12, 13, 18, 19, 25, 28]. Recent match injury incidence further suggests fast bowling was accountable for the most injuries reported, 41.6 injuries/1000 days of play [20]. Previous research has demonstrated links between fast bowling spinal kinematics and low back pain and injury [1, 40]. Furthermore, systematic reviews have concluded that specific spinal kinematics associated with fast bowling are associated with risk of low back pain [17, 25, 31]; therefore, the measurement of cricket fast bowling remains important for injury risk.

Analysis of fast bowling technique has typically been conducted in specific laboratories where optoelectronic motion analysis systems and force plates are embedded [3, 8, 24, 44, 57]. Such a set-up can provide a controlled environment with highly accurate and reliable kinetic and kinematic computation; however, there are a number of inherent limitations [26, 29, 34]. Fast bowling within a confined laboratory may result in modifications to a natural bowling action. For example, to achieve a normal run-up, either a large laboratory space or an option to capture motion outside is needed, each significantly contributing to cost. Such arrangements are likely to be beyond the scope of most non-elite clubs. Therefore, in order to make technique analysis more accessible to coaches and to enable regular monitoring of technique, alternate motion capture strategies are required.

As a surrogate for ground reaction force variables, accelerometers have been previously validated for high-impact movements including running, jumping and falling [9, 33, 39, 46, 48]. Previous research has established a strong relationship between ground reaction force (GRF) and accelerometery for running (r^2^ = 0.95) [22]. This suggests that such methods may be suitable for the detection of ground impacts associated with fast bowling. This technology may offer a solution for real-time in-field analysis of fast bowling impacts that has not previously been available to coaches.

The use of inertial sensors for human movement analysis is becoming more common with previous research documenting their use in clinical and sporting applications [6, 23, 33, 45, 50, 52, 53]. Concurrent validity for the measurement of spine kinematics using inertial sensors has yielded correlation coefficients of > 0.78 and root mean squared errors (RMSE) < 3.1° reported over 10 years ago [56]. Further enhancements and evolution of the technology and processing methods have demonstrated RMSE of < 1.9° [30, 51] and correlations of > 0.99 [51] compared to that of optoelectronic systems. Furthermore, comparisons to electromagnetic tracking systems have also shown excellent correlations (as high as r^2^ = 0.999) and small mean differences < 1° [21, 38].

Inertial sensors have a number of inherent benefits, such as not relying on line-of-sight as well as being highly portable enabling in-field data collection. A previous literature review demonstrated good reliability and validity but concluded to that their magnitude is task-specific [10]. Therefore, prior to suggesting the use of inertial sensors as an alternative for fast bowling analysis, reliability and validity should be established.

Therefore, this study aims to assess the validity and reliability of using inertial sensors to analyse three-dimensional tibial impact and spinal kinematics during cricket fast bowling.

## Methods

In order to explore the reliability and validity of an inertial sensor system, two distinct phases were completed, one for reliability and the other validity, compared to Vicon and force plate data. For clarity a table of abbreviations is provided (Table 1).

**Table 1:** List of abbreviations use in this manuscript

Abbreviation	In full
RMSE	Root mean squared error
T1	First thoracic vertebrae
L1	First lumbar vertebrae
S1	First sacral vertebrae
BFI	Back-foot impact
FFI	Front-foot impact
SCR	Shoulder counter-rotation
GRF	Ground reaction force
ICC	Intraclass correlation coefficient
SEM	Standard error of measurement
MDC	Minimal detectable change
CMC	Coefficient of multiple correlation
RMSEP	Root mean square error of prediction
ROM	Range of motion

### Participants

This study recruited 35 county-level cricket fast bowlers for the on-field reliability part of the study (mean (± SD) age 20.13 (4.62) years, height 1.84 (0.07) m and mass 80.32 (11.02) kg). A further 5 club-level fast bowlers volunteered for the validity part of the study completed in a laboratory set-up allowing full run-up (mean (± SD) age 19.33 (1.15) years, height 1.80 (0.12) m and mass 78.67 (22.30) kg).

According to county-level cricket coaches, all bowlers were categorised as fast or fast-medium bowlers. Exclusion criteria included any injury that affected their ability to bowl with maximal effort. The Bournemouth University ethics committee provided approval for this study.

### Instrumentation

#### Inertial sensor system—reliability

**Fig. 1:**
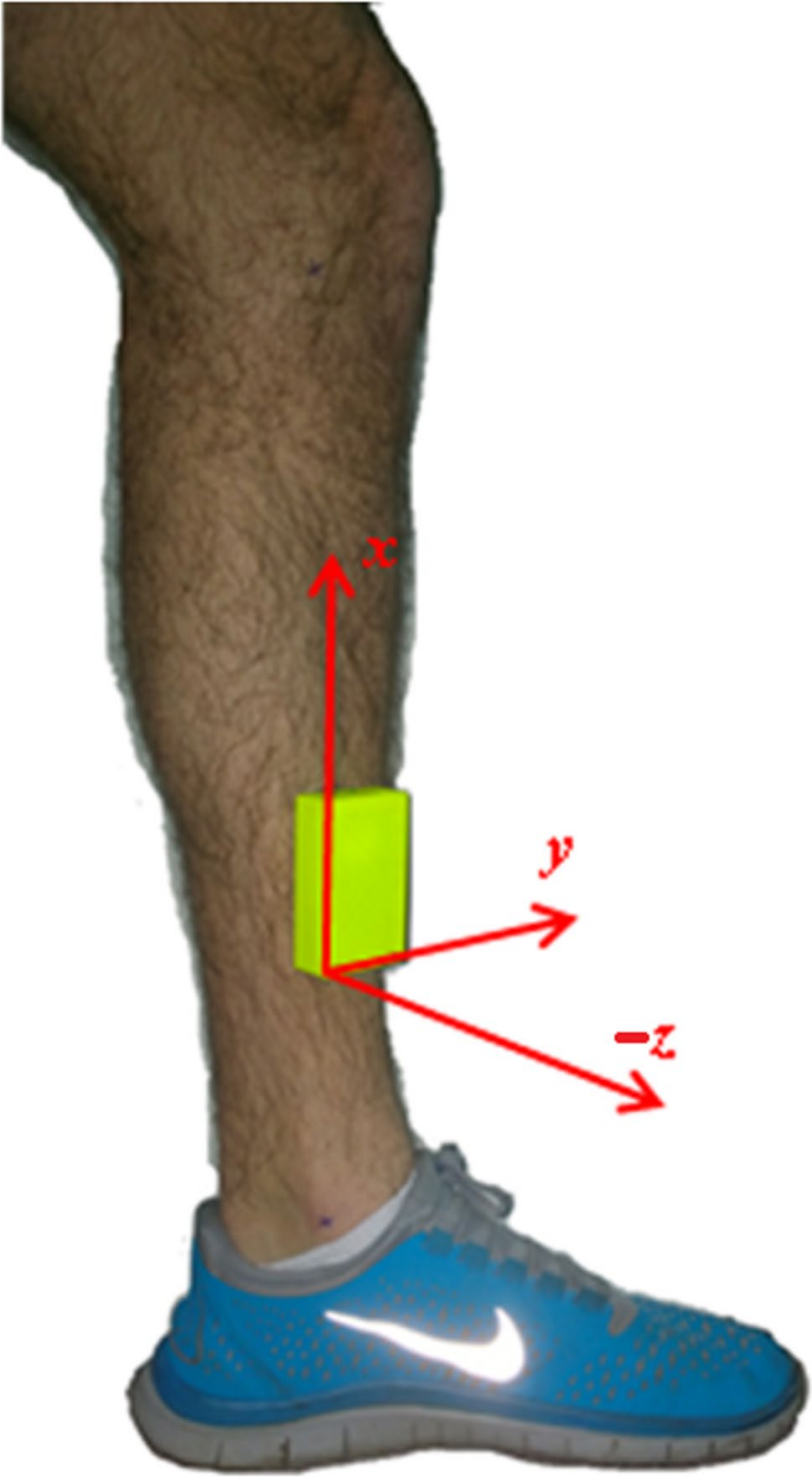
Tibial accelerometer. Axes; *x* = along-tibial axis, *y* = perpendicular to *x* along to second edge of the sensor casing and, *z* = perpendicular to *x* along the short edge of the sensor casing

The inertial sensor system consisted of synchronised tibial-mounted accelerometers and 3 trunk-mounted inertial sensors. The tibial sensors were attached to the skin over the medial tibia of each leg. This sensor housed a triaxial accelerometer with measurement range of ± 200 g, sampling at 750 Hz, and built in Bluetooth (THETAmetrix, Portsmouth, UK). This was orientated so the vertical axis aligned with the long axis of the tibia and was attached with re-enforced compressive bandage (Fig. 1, with bandage removed).

**Fig. 2:**
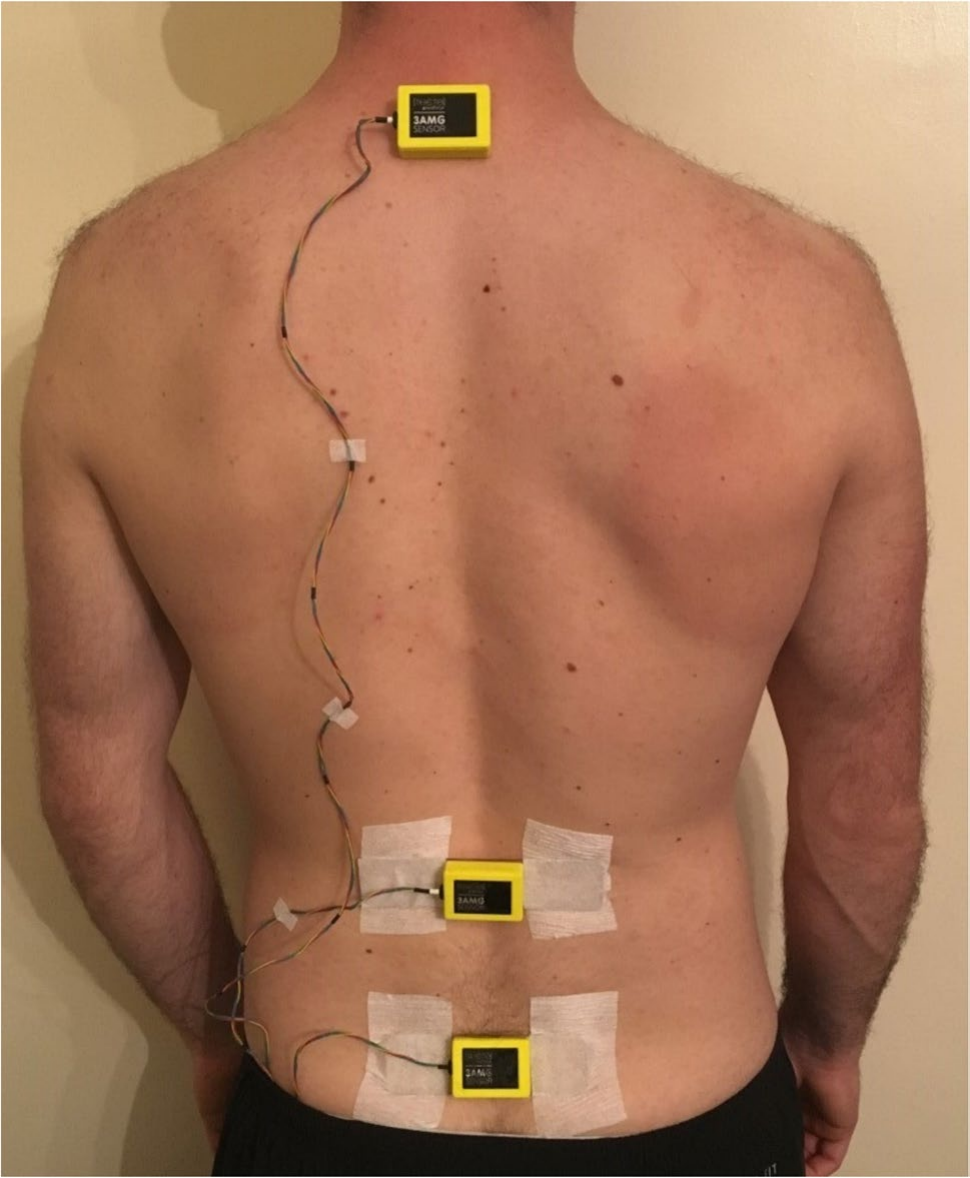
Placement of spinal inertial sensors

The three trunk-mounted sensors (THETAmetrix, Portsmouth, UK) were attached to the skin over the T1, L1 and S1 spinous processes (Fig. 2) according to the directions outlined in Field and Hutchinson [16]. These sensors housed triaxial accelerometers, gyroscopes and magnetometers, sampling at 100 Hz and sensors were wired to a small processor unit with Bluetooth (Fig. 2). Factory calibration determined sensor accuracy with average errors < 0.2° and standard deviation of error for roll and pitch of < 0.61° and heading < 2°.

#### Vicon and force plate—validity

To determine concurrent validity, the inertial sensor system was compared to a Vicon Motion Capture system, with 14 cameras, operating at 200 Hz along with two Kistler force plates (900 × 600 mm) sampling at 1000 Hz. Bowlers were attached with 39 × 14-mm retroflective markers over landmarks described by the full body plug-in-gait model (Vicon Nexus 2.7). Data from inertial sensors and Vicon were simultaneously but asynchronously captured for comparison.

### Procedure

After warming up, each bowler was instrumented as described above and completed 6 bowls with maximal effect to familiarise themselves with bowling with sensors/markers attached. Once complete, data were captured for 6 maximal effort bowls. For the reliability aspect of the study, all bowlers bowled at a right-handed batsman on grass wickets. For the validity aspects bowlers bowled into a net 5 m away from the point of ball release in a laboratory allowing a full-length run-up. If clean contact with force plates at back-foot impact (BFI) and front-foot impact (FFI) were not achieved, the trial was repeated.

### Data processing

#### Inertial sensor system—tibia

**Fig. 3:**
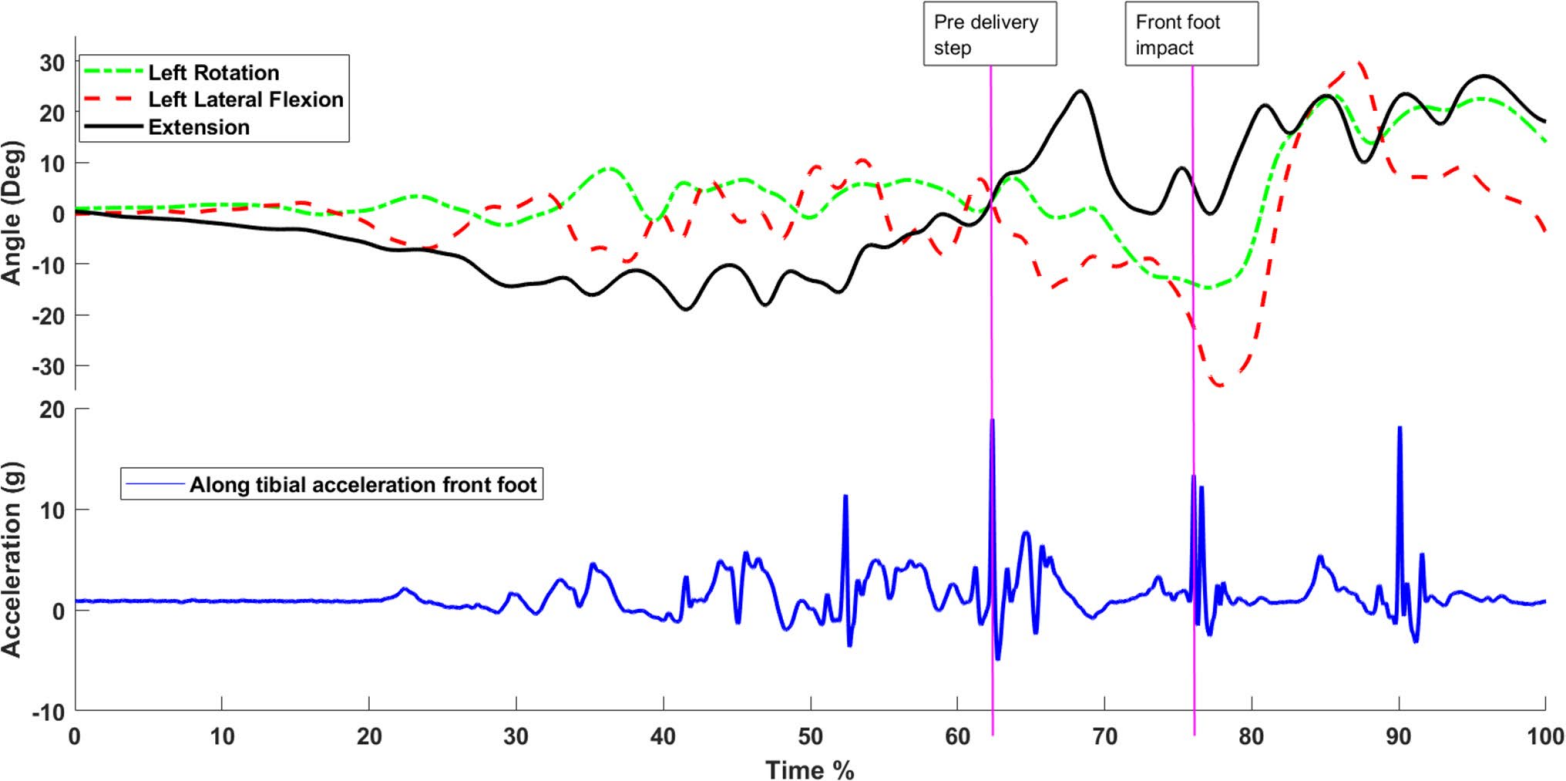
Tibial acceleration and lumbar kinematics during fast bowling

All data processing was completed in Matlab (Ed. R2012a) using bespoke algorithms. The focus of this study was on the fast bowling delivery stride defined as the final BFI to the point of FFI prior to ball release. The sensors of the inertial sensor system were synchronised, enabling the points of BFI and FFI to be determined from the along tibia impact peaks. Tibial acceleration data were low-pass filtered with a bidirectional second-order, low-pass Butterworth filter at 50 Hz. This was determined through residual analysis and used to remove high-frequency noise [39, 55]. From this data, first, the largest peak of the delivery stride for the front leg was determined as the point of FFI and the largest peak on the other leg prior to this FFI peak was identified as the BFI. Each peak was identified manually by the same investigator (Fig. 3). Tibial acceleration data were described relative to the orientation of the tibia because a lack of integrated gyroscope prevented the ability to determine the dynamic tilt angle of the sensor needed for tilt correction.

Peak tibial accelerations (*x*, *y* and *z*) and peak resultant tibial acceleration (square root of the sum of the squared accelerations for each axes) were determined for BFI and FFI. Time-to-peak tibial acceleration and time-to-peak resultant tibial acceleration for both BFI and FFI phase of the bowling stride were also determined manually as the time taken from the point of initial inflection of the acceleration peak to the peak.

#### Inertial sensor system—spinal kinematics

The trunk-mounted inertial sensors provided Euler angle orientation outputs from which three-dimensional rotation matrices for each sensor were calculated and multiplied to derive resultant orientation between 2 sensors in three dimensions. Therefore, resultant Euler angles were then extracted and used to describe the relative motion between the S1 and L1 sensors (lumbar kinematics) as previously described [5, 45, 53]. All movements were determined relative to the natural standing posture representing the initial frame of reference. Residual analysis was used to determine a 5-Hz cutoff frequency for the second-order zero-lag Butterworth filter applied to the kinematic data, to remove high-frequency noise [5, 55]. Movement-time curves between BFI and FFI (defined, as above, from the tibial-mounted sensors) were determined and time-normalised, using linear interpolation, to enable comparison of delivery strides, as is common in fast bowling literature. Lumbar kinematics during the delivery stride was reported for flexion/extension, lateral bending and rotation. Furthermore, shoulder counter-rotation (SCR) was determined by subtracting T1 orientation at BFI from T1 maximum right rotation (SCR) [34]. All data were converted to read as for right-handed bowlers therefore flexion, left lateral flexion and left rotation were defined as positive.

#### Ground reaction force from force plate for validity testing

Ground reaction force (GRF) data were used to define the delivery stride (BFI to FFI) for the validation aspects of the study. The force plate data were processed using a method similar to that described above. Raw data were low-pass filtered (as for tibia sensors), and BFI and FFI were defined as the change in GRF > 5 N for the corresponding limb. Conceptually, similar variables were determined from ground reaction force data, resulting in vertical, anteroposterior and mediolateral GRF, resultant peak GRF and time-to-peak vertical and resultant GRF variables for BFI and FFI.

#### Spinal kinematics from Vicon for validity testing

A pelvis segment was created from markers attached to the left and right anterior and posterior iliac spines. A thorax segment was constructed using markers attached to the clavicle, sternum, C7 and T10 markers. To mirror the inertial sensors, movement-time curves between BFI and FFI (defined from synchronised GRF data) were determined and time-normalised, using linear interpolation, to enable comparison of between systems. In addition, shoulder counter-rotation was defined as the orientation of the thorax at BFI subtracted from maximum rotation away from the direction of delivery.

### Statistical analysis

#### Reliability

Average measures intraclass correlation coefficients (ICC_3,k_), standard error of measurement (SEM) and minimum detectable change at the 95% confidence level (MDC) were carried out for the tibia acceleration and range of motion variables [11]. This provides a measure of consistency and variability for the peak range of motion (ROM) values only. Therefore, for the spinal range of motion data, the coefficient of multiple correlation (CMC) and root mean square error (RMSE) were also calculated between BFI and FFI to provide a measure of consistency and variability of the spinal movement behaviour across time for the whole delivery stride [15, 53]. Interpretation of reliability values is based on 0.5–0.75 moderate reliability, 0.75–0.9 good reliability and > 0.9 excellent reliability [27].

#### Validity

All tibial accelerations and ground reaction force data were normally distributed; therefore, the relationship between tibial accelerations and GRF was assessed via Pearson’s correlations. A Bonferroni correction for multiple comparisons was applied, resulting in an alpha of 0.003. Correlations were assessed on a bowl-by-bowl basis; therefore, 30 bowls were compared. Correlations were run on conceptually comparative measures such as peak vertical GRF and peak along-tibial acceleration. All peaks and time-to-peak variables were compared.

As spinal kinematics between Vicon and inertial sensors are expressed in the same metric, further comparisons were able to be carried out on this data. Pearson’s correlations were conducted as well as mean bias (mean difference between measurements) and root mean square error of prediction (RMSEP) were calculated [58].

## Results

### Inertial sensor system reliability—tibial accelerations

A typical graph of along-tibial acceleration is presented in Fig. 3. The graph illustrates the identifiable phases of run-up, pre-delivery step, front-foot impact and follow-through demarcated by respective impact peaks. Mean (SD) tibial accelerations for BFI and FFI can be seen in Table 2.

**Table 2:** Mean (± SD) and reliability of tibial acceleration at back-foot and front-foot impact during fast bowling

	Back-foot impact				Front-foot impact			
	Mean (± SD)	ICC	SEM	MDC	Mean (± SD)	ICC	SEM	MDC
Tibial acceleration								
Peak tibial acc *x* (g)	12.42 (5.57)	0.95	1.22	3.38	25.91 (11.31)	0.97	1.96	5.43
Peak tibial acc *y* (g)	4.29 (3.7)	0.93	1.01	2.80	12.42 (8.21)	0.93	2.19	6.07
Peak tibial acc *z* (g)	15.85 (8.76)	0.96	1.80	4.99	20.31 (11.91)	0.95	2.79	7.73
Resultant tibial acc (g)	20.11 (7.80)	0.97	1.42	3.94	35.17 (15.26)	0.95	3.31	9.17
Time-to-peak tibial acc *x* (ms)	25.47 (11.10)	0.73	5.78	16.02	20.92 (10.39)	0.84	4.10	11.36
Time-to-peak resultant tibial acc (ms)	54.59 (21.80)	0.90	7.30	20.23	58.29 (13.48)	0.53	9.24	25.61

*ICC*, intraclass correlation coefficient; *SEM*, standard error of measurement; *MDC*, 95% minimum detectable change; *Acc*, acceleration; *g*, gravity; *ms*, milliseconds; *SD*, standard deviation.

Peak tibial acceleration (all planes) and resultant tibial acceleration demonstrated excellent reliability at BFI and FFI (Table 2). Time-to-peak and time-to-resultant peak demonstrated moderate to excellent reliability, depending on the specific foot (Table 1). SEM and MDC measures were low (Table 2) suggesting that with 95% confidence, an alteration in tibial acceleration greater than 3.4 g for along-tibial acceleration represents a change greater than the natural variation observed during repeated bowling. Likewise, 16.0 ms is the threshold for true change beyond natural variation at the 95% confidence level for along-tibial time-to-peak impact. Furthermore, a change greater than 5.4 g or 11.4 ms for the corresponding variables at front-foot impact represents change greater than natural bowling variation.

### Inertial sensor system reliability—spinal kinematics

A typical lumbar kinematics graph is presented in Fig. 3. Mean (SD) spinal orientations and resultant ROM for the delivery stride can be seen in Table 3.

**Table 3:** Reliability of fast bowling spinal range of motion between back-foot and front-foot impact

	ICC	SEM (°)	MDC (°)	CMC	RMSE (°)
Shoulder counter-rotation	0.72	2.66	7.37		
Lumbar flexion	0.93	4.02	11.14	0.63	3.93
Lumbar lateral flexion	0.64	4.46	12.36	0.71	2.92
Lumbar rotation	0.67	4.82	13.36	0.70	4.32

*ICC*, intraclass correlation coefficient; *SEM*, standard error of measurement; *MDC*, 95% minimal detectable change; °, degrees; *CMC*, coefficient of multiple correlation; *RSME*, root mean square error.

All kinematic variables demonstrated moderate to excellent reliability and small SEM and MDC values thus demonstrating minimal intra-individual variability for repeated fast bowling (Table 3). The reliability of movement-time curve as measured by CMCs was moderate, and RMSEs were small demonstrating moderate to good reliability over the whole delivery stride (Table 3).

### Inertial sensor system validity—GRF

Significant pairwise correlations (p < 0.003) were determined for 79% of the comparisons between acceleration and GRF variables (Table 4). Except for time-to-peak resultant acceleration (r = 0.640), good to excellent correlations were observed for all variables.

**Table 4:** Comparison and correlation of mean tibial acceleration and ground reaction force

GRF variable	Mean (± SD)	Accelerometer variable	Mean (± SD)	r
Back-foot impact				
Vertical peak GRF (N)	1738.4 (391.2)	Along-tibial peak acceleration (g)	14.1 (6.6)	0.974*
Anterior–posterior peak GRF (N)	845.8 (138.1)	Anterior–posterior peak acceleration (g)	11.7 (6.4)	0.977*
Mediolateral peak GRF (N)	254.2 (150.8)	Mediolateral peak acceleration (g)	3.5 (3.2)	0.966*
Resultant peak GRF (N)	1875.5 (379.8)	Resultant peak acceleration (g)	20.4 (9.4)	0.968*
Time-to-peak vertical GRF (ms)	30.4 (16.8)	Time-to-peak along-tibial acceleration (ms)	25.8 (8.5)	0.979*
Time-to-peak resultant GRF (ms)	34.5 (15.4)	Time-to-peak resultant acceleration (ms)	22.4 (9.4)	0.767
Front-foot impact				
Vertical peak GRF (N)	3072 (921.9)	Along-tibial peak acceleration (g)	30.9 (14.4)	0.871*
Anterior–posterior peak GRF (N)	604.6 (587.3)	Anterior–posterior peak acceleration (g)	23.5 (8.4)	0.860*
Mediolateral peak GRF (N)	405.2 (388.0)	Mediolateral peak acceleration (g)	16.7 (8.4)	0.878*
Resultant peak GRF (N)	3206.7 (965.1)	Resultant peak acceleration (g)	46.4 (20.8)	0.946*
Time-to-peak vertical GRF (ms)	15.7 (10.1)	Time-to-peak along-tibial acceleration (ms)	18.2 (3.2)	0.772
Time-to-peak resultant GRF (ms)	15.8 (10.1)	Time-to-peak resultant acceleration (ms)	16.6 (2.8)	0.640

*Denotes p < 0.003

*GRF*, ground reaction force; *N*, Newtons; *ms*, milliseconds; *g*, gravity; *SD*, standard deviation; *r*, Pearson’s correlation coefficient.

### Inertial sensor system validity—spinal kinematics

Moderate to excellent pairwise correlations were determined for lumbar kinematic variables at both BFI and FFI with mean bias estimates highlighting inertial sensor data overestimated kinematics between 1.9 and 4.0° (Table 5). Lumbar rotation at BFI resulted in only a moderate correlation, and at FFI was significantly larger using Vicon (p = 0.029). Consequently, root mean square error of prediction (RMSEP) ranged from 0.3 to 1.5°.

**Table 5:** Comparison and correlation of mean spinal kinematics, mean bias and RMSEP between inertial sensors and optoelectronic motion analysis at back and front-foot impact

Variable	Optoelectronic (° ± SD)	IMU (° ± SD)	r	Mean bias (°)	RMSEP (°)
Shoulder counter-rotation	24.9 (7.7)	24.0 (7.7)	0.948*	− 0.9	0.3
Lumbar flexion at BFI	5.7 (5.6)	7.5 (4.7)	0.986*	1.9	0.5
Lumbar lateral flexion at BFI	5.8 (2.1)	9.8 (6.6)	0.949*	4.0	1.2
Lumbar Rotation at BFI	10.3 (6.4)	12.1 (9.9)	0.612	1.8	0.5
Lumbar flexion at FFI	13.6 (8.8)	17.3 (5.0)	0.958*	3.6	1.1
Lumbar lateral flexion at FFI	10.8 (10.9)	13.9 (7.2)	0.954*	3.2	0.9
Lumbar rotation at FFI	21.2 (7.5)	16.1 (7.3)	0.846*	− 5.1	1.5

*Denotes p < 0.003

*BFI*, back-foot impact; *FFI*, front-foot impact; °, degrees, *SD*, standard deviation; *IMU*, inertial measurement unit; *RMSEP*, root mean squared error of prediction; *r*, Pearson’s correlation coefficient. Flexion, left lateral flexion and left rotation were defined as positive.

## Discussion

The aim of this study was to determine whether inertial sensors and accelerometers were able to quantify tibial impact and lumbar kinematics during the delivery stride of cricket fast bowlers. To the author’s knowledge, this is the first study of its kind, and it demonstrates that inertial sensor and accelerometers can offer an on-field method to explore cricket fast bowling. Previous research into cricket fast bowling has concentrated on the use of optoelectronic camera systems and force plates to determine GRF and spinal kinematics [3, 8, 14, 24, 26, 29, 34, 41, 43, 57]. Whilst such systems undoubtedly offer unrivalled motion capture capability, they are expensive and require a specific data capture environment like a laboratory with adequate space to afford full run-up [8, 34, 57].

### Tibial acceleration

Previous literature has reported limb impacts relating to fast bowling with a strong reliance on the use of force plates [8, 32, 57]. Force plates offer a reliable, comparable and accurate method for measuring limb impacts, and are believed to represent the gold standard in research. However, force plates are costly and are commonly constrained to a laboratory. Moreover, results pertaining to different surface conditions are not possible with force plates. This study proposed an alternate method, and the results suggest that a tibial-mounted accelerometer is a valid method of measuring tibial accelerations relating to foot impact during real-time, in-field cricket fast bowling. The results from this study demonstrate that tibial accelerometer values correlated well with comparative metrics from the force plate. This was particularly strong for magnitude related variables demonstrating that a tibial accelerometer is a strong surrogate measure of GRF. A method as outlined above could offer coaches a simple and cost-effective way to monitor limb impact during fast bowling and has the advantage of being applicable to any surface or environment, i.e. nets and indoor.

This study demonstrates that accelerometers for measuring impact measurement are reliable for repeated measures. This is critical for coaches wishing to monitor change associated with technique modification or associated with injury. Previous studies investigating reliability of tibial accelerometers report ICC values of 0.64–0.97 for walking and 0.82 for running, demonstrating that the reliability of tibial accelerometers for the analysis of fast bowling impacts is similar to other tasks [37, 49]. Small SEM and MDC values provide the coaching team with a level of confidence for interpreting true change in bowling technique suggesting such a technique is sensitive to detecting subtle changes in bowling which are beyond natural variation experienced during repeated bowling.

One novel finding of the present study is the values of time-to-peak along-tibial acceleration (20.92 (± 10.39) ms). Previous studies have suggested time-to-peak vertical GRF values between 26 and 90 ms [8, 24, 34, 57]. The inherent difference is likely to be due to the methodological differences between the two methods. GRF studies have reported vertical GRF; however, the present study was unable to correct acceleration to yield true vertical, anteroposterior and mediolateral acceleration. Due to the absence of any other sensing elements in the tibial-mounted accelerometer (i.e. a gyroscope), the correction for the tilt of the sensor on the tibia was not possible. Therefore, at foot impact, it is unlikely that the tibial sensor is vertical and therefore represents an axis-oriented along the tibia. Despite these differences, the values for time-to-peak are similar to those reported in the literature. Further novelty from this study includes the reporting of time-to-peak at BFI. These values for BFI have not been reported for GRF literature either; therefore, they are a new contribution to the understanding of cricket fast bowling and enable further exploration of the relationship between fast bowling impacts and musculoskeletal injury, which have been described as ‘rate-dependant’ [7, 47]. It is possible to resolve the issue with sensor orientation on the tibia by the integration of a triaxial gyroscope from which the sensor orientation at impact can be derived and corrected for.

### Spinal kinematics

Previous studies have demonstrated the concurrent validity of using inertial sensors for spinal ROM [2] with excellent correlation being reported. However, this is the first investigation into the ability of inertial sensors to be able to measure lumbar kinematics during cricket fast bowling and therefore extends our understanding of the capabilities of in-field measurement. The findings pertaining to lumbar range of motion during cricket fast bowling are comparable to those in the published literature (Table 6). The excellent correlations (except for lumbar rotations which were moderate to good) between the optoelectronic gold standard and the inertial sensors demonstrate that inertial sensor offer a valid way of measuring lumbar kinematics and SCR during fast bowling.

**Table 6:** Three-dimensional spinal kinematics (± SD) reported in previous research and this study

Authors	Participants	Spinal segment analysed	Bowling phase analysed	Flexion (°)	Extension (°)	Left lateral flexion (°)	Left rotation (°)
Current Study	35	S1-L1	BFI-FFI	21 ± 8	14 ± 14	20 ± 8	14 ± 7
Bayne et al. 2016	13 12	L5-L1	FFC-BR	20 ± 4 21 ± 5		11 ± 4 12 ± 3	4 ± 2 5 ± 2
Crewe et al. 2013	13 18 8	S1-L1	FFI-BR			10 ± 4 12 ± 3 11 ± 3	
Stuelcken et al. 2010	14 12	S1-T1	BFI-BR	27 ± 12 29 ± 10	14 ± 9 13 ± 9	42 ± 6 38 ± 6	26 ± 6 27 ± 6
Ferdinands et al. 2009	21	S2-T10	BFI-FFI	38 ± 8	6 ± 2	16 ± 11	19 ± 2
Ranson et al. 2009	14	S1-T10	BFI-FFI		0 ± 7	34 ± 7	29 ± 9
Ranson et al. 2008	50	S1-T10	BFI-BR		9 ± 6	34 ± 7	32 ± 8
Burnett et al. 1998	20	S2-L1	BFI-FFI	48	10	30	11

°, degrees; *BFI*, back-foot impact; *FFC*, front-foot contact; *FFI*, front-foot impact; *BR*, ball release.

The greatest differences in range were observed for lumbar rotation. These small differences (< 5.1°) may be due to different definitions of the thoracic segment and skin movement artefact. The definition of the thoracic segment involved motion up to T10, and therefore, the resulting lumbar spine is slightly longer for the optoelectronic model compared to the inertial sensor model potentially explaining the difference in these measurements. Moreover, as the sacral sensor is positioned over S1, where there is potential for a lot of skin movement, confounded by the ballistic action of the fast bowling. Attempts to counteract this were made in the study through reinforced attachments, and the contribution of this artefact is not clear. Future studies should work to explore the mechanism behind the difference in rotation measured by the inertial sensors.

Optoelectronic motion analysis of fast bowling spinal kinematics has reported ICCs of 0.74–0.98 and SEM 1–17° [35, 36]. The results of this study show that inertial sensors offer similar levels of repeated measures reliability as those seen with this gold standard. Lower ICCs were seen in lumbar lateral flexion and rotation (0.63–0.67) suggesting slightly greater variability in peak values for these planes of motion. However, the SEM was similar across planes, less than 5° highlighting small repeated measures differences associated with repeated peak values. These values are constructed of error associated with the sensor combined with the human-sensor interaction, as well as the natural variability of this particular task (biological variability). SEMs recorded in this study are in line with those reported by optoelectronic systems during bowling [35, 36] further suggesting the validity of inertial sensors for fast bowling analysis.

The MDC values are a way to provide the coach or clinician with a guide to the natural variability between repeated bowls. From the study, a change in range of movement greater than 13° could be interpreted as true change beyond the realm of movement variability. However, it is important to note that MDC values are different across movement planes. This is the first time values have been reported in the literature for inertial sensors, and such values are important to clinicians and coaches who may be monitoring alterations in bowling technique either through coaching interventions or as a result of pain [54]. It is important to identify that these values are drawn from the peak values observed during bowling and therefore do not provide an understanding of the similarities in movement behaviour across the bowling stride.

In addition to peak estimates, this study calculated CMC values demonstrating moderate to good reliability for kinematic curves across time suggesting movement patterns were consistent (Table 3). These findings suggest that fast bowlers were able to reproduce similar movement patterns and that inertial sensors were able to capture this reliably with small degrees of variation. Previous studies have demonstrated consistent kinematics over long bowling spells, suggesting that the motion of fast bowling is one associated with high levels of internal consistency [4]. To date, only one previous study has reported CMC values which were slightly higher than those observed in this study (CMCs > 0.89, [5]). This may be due to the number of repeated bowls used, which was three compared to six for the current study, or greater movement variability demonstrated by the participants in the current study (Table 6).

### Conclusion

To the author’s knowledge, this is the first study to investigate the validity and reliability of inertial sensors to measure tibial accelerations and spinal kinematics during ‘in-field’ fast bowling. This study demonstrates inertial sensors offer moderate to excellent estimates of reliability and validity when used for collecting lumbar kinematics and tibial impacts during cricket fast bowling and that the resultant measurements were similar to those previously reported and concurrently collected.
